# A Replication Study of GWAS-Derived Lipid Genes in Asian Indians: The Chromosomal Region 11q23.3 Harbors Loci Contributing to Triglycerides

**DOI:** 10.1371/journal.pone.0037056

**Published:** 2012-05-18

**Authors:** Timothy R. Braun, Latonya F. Been, Akhil Singhal, Jacob Worsham, Sarju Ralhan, Gurpreet S. Wander, John C. Chambers, Jaspal S. Kooner, Christopher E. Aston, Dharambir K. Sanghera

**Affiliations:** 1 Department of Pediatrics, College of Medicine, University of Oklahoma Health Sciences Center, Oklahoma City, Oklahoma, United States of America; 2 Section of Cardiology, Hero Dayanand Medical College and Hospital Heart Institute, Ludhiana, Punjab, India; 3 Department of Epidemiology and Biostatistics, Imperial College London, London, United Kingdom; 4 National Heart and Lung Institute, Imperial College London, London, United Kingdom; 5 Harold Hamm Diabetes Center, University of Oklahoma Health Sciences Center, Oklahoma City, Oklahoma, United States of America; Oklahoma Medical Research Foundation, United States of America

## Abstract

Recent genome-wide association scans (GWAS) and meta-analysis studies on European populations have identified many genes previously implicated in lipid regulation. Validation of these loci on different global populations is important in determining their clinical relevance, particularly for development of novel drug targets for treating and preventing diabetic dyslipidemia and coronary artery disease (CAD). In an attempt to replicate GWAS findings on a non-European sample, we examined the role of six of these loci (*CELSR2-PSRC1-SORT1* rs599839; *CDKN2A-2B* rs1333049; *BUD13-ZNF259* rs964184; *ZNF259* rs12286037; *CETP* rs3764261; *APOE-C1-C4-C2* rs4420638) in our Asian Indian cohort from the Sikh Diabetes Study (SDS) comprising 3,781 individuals (2,902 from Punjab and 879 from the US). Two of the six SNPs examined showed convincing replication in these populations of Asian Indian origin. Our study confirmed a strong association of *CETP* rs3764261 with high-density lipoprotein cholesterol (HDL-C) (p = 2.03×10^−26^). Our results also showed significant associations of two GWAS SNPs (rs964184 and rs12286037) from *BUD13-ZNF259* near the *APOA5-A4-C3-A1* genes with triglyceride (TG) levels in this Asian Indian cohort (rs964184: p = 1.74×10^−17^; rs12286037: p = 1.58×10^−2^). We further explored 45 SNPs in a ∼195 kb region within the chromosomal region 11q23.3 (encompassing the *BUD13-ZNF259, APOA5-A4-C3-A1, and SIK3* genes) in 8,530 Asian Indians from the London Life Sciences Population (LOLIPOP) (UK) and SDS cohorts. Five more SNPs revealed significant associations with TG in both cohorts individually as well as in a joint meta-analysis. However, the strongest signal for TG remained with *BUD13-ZNF259* (rs964184: p = 1.06×10^−39^). Future targeted deep sequencing and functional studies should enhance our understanding of the clinical relevance of these genes in dyslipidemia and hypertriglyceridemia (HTG) and, consequently, diabetes and CAD.

## Introduction

Dyslipidemia, with low levels of high-density lipoprotein cholesterol (HDL-C) and high levels of low-density lipoprotein cholesterol (LDL-C) and triglycerides (TG), is a well established risk factor for coronary artery disease (CAD) and a significant cause of mortality in individuals with type 2 diabetes (T2D) [1]. The risk of developing CAD is 2–3 times higher in diabetic males and 4–5 times higher in diabetic females compared to male and female non-diabetics [2]. There is considerable ethnic difference in the prevalence and progression of T2D and CAD; the incidences of these diseases are about 3–5 times higher in Asian Indians compared to Euro-Caucasians [3]. Lipid levels are widely measured in clinical practice and are used as therapeutic targets for prevention and treatment of CAD especially in patients with diabetes [4]. Recent genome-wide association scans (GWAS) and meta-analysis studies in European populations have identified common variants in many genes, including previously known loci that are potentially involved in lipid regulation [5]–[8]. High heritability (40% to 60%) of lipid traits and strong association signals among common variants in these genes involved in lipid metabolism provide a strong rationale to search for causal variants that may uncover novel pathways crucial for lipid regulation and eventually lead to treatment or prevention of CAD [9], [10]. Replication of GWAS signals in different ethnic groups is important as the frequency of the susceptible alleles at these loci may vary significantly between world populations [11]. Also, these studies can help identify population-specific environmental factors controlling disease risk or protection associated with specific demographic and cultural histories [11]. In particular, replication of GWAS loci associations will have more relevance in population groups with high disease burdens such as Asian Indians [12].

A few studies have reported associations of these novel loci with lipid traits in Asian Indian immigrants living in the UK [6], [13], [14]. The present investigation was carried out to examine the role of six of the most strongly associated and extensively replicated GWAS loci (*CELSR2-PSRC1-SORT1* rs599839; *CDKN2A-2B* rs1333049; *BUD13-ZNF259* rs964184; *ZNF259* rs12286037; *CETP* rs3764261; *APOE-C1-C4-C2* rs4420638) (summarized in Table 1) in our Asian Indian cohort from the Sikh Diabetes Study (SDS) [15]. By further expanding our search around a ∼195 kb region within the chromosomal region 11q23.3 surrounding *BUD13-ZNF259, APOA5-A4-C3-A1,* and *SIK3* gene clusters in 8,530 Asian Indian individuals, we not only confirmed the strongest signal associating rs964184 (from the inter-genic region of *BUD13-ZNF259*) with TG, but also discovered strong association in several other SNPs in this region using single-SNP association and haplotype analysis.

**Table 1:** Details of the investigated loci.

Gene	SNP	Chr. Position	Trait	Allele	Ref. Freq.	SDSFreq.	p-value	Effect	Population/Study	References
*CELSR2-PSRC1-SORT1*	rs599839	Chr 1:109623689	LDL	A/**G** *	0.21	0.26	1.7×10^−15^	−0.1	Caucasian	Sandhu et al, 2008, Lancet
			LDL	**A**/G	0.77		6.1×10^−33^	5.5	FUSION	Willer et al, 2008, Nat Genet
			LDL	A/**G**	0.08		3.1×10^−11^	−4.7	Japanese	Nakayama et al, 2009, J Med Genet
*CDKN2A-2B*	rs1333049	Chr 9:22115503	CAD	**C**/G	0.47	0.50	1.2×10^−13^	0.4	WTCCC	Burton et al, 2007, Nature
			MI	**C**/G	0.50		0.02	0.1	Hispanics	Qi et al, 2011, Circulation
*BUD13-ZNF259*	rs964184	Chr 11:116648917	TG	C/**G**	0.14	0.20	4×10^−62^	0.3	FHS	Kathiresan et al, 2008, Nat Genet
			TG	C/**G**	0.13		7×10^−240^	16.9	Caucasian	Teslovich et al, 2010, Nature
			TG	C/**G**	0.14		5.4×10^−24^	–	Caucasian	Johansen et al, 2010, Nat Genet
			HDL	C/**G**	0.14		1×10^−12^	−0.2	FHS	Kathiresan et al, 2008, Nat Genet
*ZNF259*	rs12286037	Chr 11:116157417	TG	**C**/T	0.94	0.96	1.0×10^−26^	25.8	FUSION	Willer et al, 2008, Nat Genet
*CETP*	rs3764261	Chr 16:55550825	HDL	**C**/A	0.69	0.64	2.3×10^−57^	3.5	FUSION	Willer et al, 2008, Nat Genet
			HDL	C/**A**	0.32		7×10^−380^	3.4	Caucasian	Teslovich et al, 2010, Nature
*APOE-C1-C4-C2*	rs4420638	Chr 19:50114786	LDL	**G**/A	0.82	0.89	3.0×10^−43^	6.6	FUSION	Willer et al, 2008, Nat Genet
			LDL	G/**A**	0.16		4×10^−27^	0.3	FHS	Kathiresan et al, 2008, Nat Genet
			LDL	G/**A**	0.17		9×10^−147^	7.1	Caucasian	Teslovich et al, 2010, Nature

* fonts in bold indicate risk allele.

## Results

Table 2 summarizes and compares the general characteristics of the Punjabi and US cohorts used in this investigation. The US cohort was younger and had an earlier onset of T2D (42.4±18.9 years) compared to the Punjabi cohort (47.6±11.1 years). Diabetics in the Punjabi cohort had poorer glycemic control showing significantly higher fasting blood glucose (FBG ) levels by ∼28 mg/dL (p = 0.002), and had a significantly higher waist to hip ratio (WHR) (by 5 percentage points) (p = 0.001), compared to the US cohort. As expected, T2D cases had significantly higher fasting TG (p<0.0001) and significantly lower HDL-C (p<0.0001) compared to normoglycemic (NG) controls. No SNP genotype deviated significantly from Hardy-Weinberg expectations (HWE) in the NG controls. Of these SNPs, no variant revealed any significant evidence of association with T2D or CAD in this population after adjusting for age, gender, and body mass index (BMI) (data not shown).

**Table 2:** Clinical characteristics of study subjects (Mean ± SD).

	Punjabi Cohortn = 2,902	p valueΨ	US Cohortn = 879	p valueΨ	Combined Cohortsn = 3,781	p valueΨ
Age (yrs.)	53.8±13.0		48.0±13.5		52.5±13.3	
% Males	55.8		51.7		54.9	
Age at Diagnosis (yrs.)	47.6±11.1		42.4±18.9		47.4±11.6	
Duration of Diabetes (yrs.)	7.7±6.8		6.8±7.2		7.6±6.8	
BMI (kg/m^2^)	26.5±5.0		26.8±4.3		25.3±7.3	
Waist (cm)	93.1±12.1		92.5±13.4		92.9±12.4	
WHR	0.95±0.07		0.90±11.0*		0.94±0.10	
Blood Pressure (mm/Hg)						
Systolic	137.6±23.6		129.6±20.9		135.7±23.2	
Diastolic	81.8±12.8		81.3±11.2		81.7±12.5	
FBG (mg/dL)						
Non-Diabetic	95.3±12.1	<0.0001	95.7±10.1	<0.0001	95.4±11.5	<0.0001
Diabetic	179.9±73.7		152.4±46.6**		177.2±72.0	
2 h glucose (mg/dL)						
Non-Diabetic	105.2±19.2	<0.0001	109.3±17.1	<0.0001	107.0±18.4	<0.0001
Diabetic	190.1±73.1		239.6±74.6¥		201.0±76.2	
Cholesterol (mg/dL)						
Non-Diabetic	171.2±54.2	0.283	188.8±45.0	0.001	177.6±52.1	0.047
Diabetic	173.6±48.4		174.7±45.9		173.7±48.1	
Triglyceride (mg/dL)						
Non-Di1abetic	147.8±71.8	0.001	121.8±71.1	<0.0001	137.0±73.2	<0.0001
Diabetic	159.3±84.1		167.5±94.8		160.2±85.3	
HDL Cholesterol (mg/dL)						
Non-Diabetic	38.1±15.4	0.001	42.2±14.2	0.001	39.5±15.2	<0.0001
Diabetic	36.1±12.8		37.8±16.4		36.3±13.1	
LDL Cholesterol (mg/dL)						
Non-Diabetic1	100.9±37.2	0.522	122.8±34.8	0.003	108.1±38.2	<0.0001
Diabetic	99.9±37.7		110.6±38.0		100.7±37.8	
NG† (%)	43.5		64.7		48.2	
T2D‡ (%)	51.9		16.0		43.6	
CHD (%)	27.3		2.5		21.5	
IGT/IFG•• (%)	4.6		19.2		8.0	

† Normoglycemic;

‡ Type II Diabetes;

•• Impaired glucose tolerance, Impaired fasting glucose,

Ψ Difference between non-diabetic and diabetic.

* p<0.001;

** p = 0.002;

¥ p = 0.02 (showing significant difference in the Punjabi and US cohorts).

### Association of CETP Variant with HDL and Triglyceride Levels

We investigated the association of all six variants with quantitative traits associated with obesity, blood glucose and serum lipids in NG and T2D individuals from both the Punjabi and US cohorts. None of the investigated SNPs showed any significant association with obesity (BMI, WHR), or glucose traits (FBG, 2 h glucose, fasting insulin, insulin resistance [HOMA-IR] and β-cell function [HOMA-B]) (data not shown). Multiple linear regression analysis revealed a strongly significant association of the ‘A’ allele of rs3764261 *(CETP)* with HDL-C in the NG (β = 0.09, p = 1.14×10^−6^), T2D (β = 0.07, p = 0.014) and combined (NG+T2D) (β = 0.09, p = 1.21×10^−4^) groups in the Punjabi cohort was observed. Similar strong association of this SNP with HDL-C was seen in the NG (β = 0.11, p = 0.006) and NG+T2D (β = 0.10, p = 1.72×10^−9^) groups from the US cohort (Tables 3, 4). Further meta-analysis using the Punjabi and US cohorts revealed a strong association of this variant with HDL-C in both fixed-effect (β = 0.14, p = 2.03×10^−26^) and random-effect (β = 0.15, p = 4.84×10^−4^) models. Interestingly, the same ‘A’ allele carriers of *CETP* also showed a significant decrease in TG (β = −0.12, p = 1.02×10^−4^) in the T2D Punjabi cohort (Table 3).

**Table 3:** Association of SNPs with lipid traits in Punjabi Cohort.

	NG Controls								T2D Cases								Combined (NG Controls + T2D Cases)							
	β	p-value	β		p-value	β		p-value	β	p-value	β		p-value	β		p-value		β		p-value	β	p-value	β	p-value
***BUD13-ZNF259*** ** rs964184**	log additive		dominant			recessive			log additive		dominant			recessive				log additive			dominant		recessive	
TG (mg/dL)	0.10	**0.001**	0.11		**0.003**	0.20		**0.011**	0.16	**9.63×10** ^−**7**^	0.13	**3.09×10** ^−**5**^		0.13	**6.94×10** ^−**5**^			0.15	**5.94×10** ^−**10**^		0.14	**3.52×10** ^−**8**^	0.11	**6.01×10** ^−**6**^
***ZNF259*** ** rs12286037**	log additive		dominant			recessive			log additive		dominant			recessive				log additive			dominant		recessive	
TG (mg/dL)	0.02	0.487	0.02		0.534	–			0.09	**0.004**	0.09		**0.005**	0.03		0.326		0.07		**0.003**	0.07	**0.003**	0.02	0.331
***CETP*** ** rs3764261**	log additive		dominant			recessive			log additive		dominant			recessive				log additive			dominant		recessive	
TG (mg/dL)	−0.02	0.546	−0.02	0.594		−0.02	0.654		−0.12	**1.02×10** ^−**4**^	−0.12	**1.90×10** ^−**4**^		−0.08		**0.013**		−0.08	**0.002**		−0.08	**0.002**	−0.04	0.080
HDL-C (mg/dL)	0.09	**1.14×10** ^−**6**^	0.10	**1.32×10** ^−**4**^		0.15	**6.71×10** ^−**5**^		0.07	**0.014**	0.04	0.136		0.08		0.007		0.09	**1.21×10** ^−**4**^		0.06	**0.011**	0.09	**6.31×10** ^−**5**^

Table 3a only contains most significant SNPs associated with lipid traits, details of the remaining SNPs can be found in online Table S1a.

**Table 4:** Association of SNPs with lipid traits in US Cohort.

	NG Controls						Combined (NG Controls + T2D Cases)							
	β	p-value	β	p-value	β	p-value	β		p-value	β	p-value	β	p-value	
***BUD13-ZNF259*** ** rs964184**	log additive		dominant		recessive		log additive			dominant		recessive		
TG (mg/dL)	0.12	**0.005**	0.13	**0.002**	**0.03**	0.484	0.18	**2.46×10^−5^**		0.19	**1.12×10^−5^**	0.08		0.058
***ZNF259*** ** rs12286037**	log additive		dominant		recessive		log additive			dominant		recessive		
Cholesterol (mg/dL)	0.11	**0.009**	0.11	**0.014**	0.06	0.140	0.18		**3.58×10^−5^**	0.17	**1.09×10^−4^**	0.10	**0.030**	
TG (mg/dL)	0.07	0.087	0.07	0.102	0.04	0.374	0.14		**0.002**	0.13	**0.002**	0.06	0.162	
***CETP*** ** rs3764261**	log additive		dominant		recessive		log additive			Dominant		recessive		
Cholesterol (mg/dL)	0.09	**0.040**	0.09	0.051	0.06	0.177	0.03		**0.018**	0.04	**0.025**	0.04	0.109	
HDL-C (mg/dL)	0.11	**0.006**	0.09	**0.023**	0.09	**0.024**	0.10		**1.72×10^−9^**	0.11	**1.96×10^−6^**	0.14	**2.60×10^−6^**	

Table 3b only contains most significant SNPs associated with lipid traits, details of the remaining SNPs can be found in online Table S1b.

### Association of BUD13-ZNF259 Variants with Triglyceride Levels

A strong and consistent association of an inter-genic variant near *BUD13-ZNF259* (rs964184) with TG in both the Punjabi and US cohorts in all additive, dominant, and recessive genetic models, even after controlling for covariates of age, gender, BMI and disease status, where necessary. As shown in Table 3 and 4, TG levels were consistently raised among minor ‘G’ risk allele carriers in the NG group in Punjabi (β = 0.10, p = 0.001) and US (β = 0.12, p = 0.005) cohorts, the T2D group in the Punjabi (β = 0.16, p = 9.63×10^−7^), and in the NG+T2D groups in the Punjabi (β = 0.15, p = 5.94×10^−10^) and US (β = 0.19, p = 1.12×10^−5^) cohorts. Moreover, the effect sizes indicated by regression coefficients (β) were consistently higher in T2D cases compared to NG controls (e.g. for rs964184, β = 0.16; p = 9.63×10^−7^ in T2D cases vs. β = 0.10, p = 0.001 in NG controls). A similar significant increase in VLDL-C was seen among the NG and T2D groups from the Punjabi and US cohorts (data not shown). The association of this variant with TG also was statistically significant in meta-analysis for both the fixed-effect (β = 0.16, 1.74×10^−17^) and random-effect (β = 0.16, 1.74×10^−17^) models (Table 5). The other intronic variant (rs12286037) in *ZNF259* was also strongly associated with TG in the Punkabi T2D group (β = 0.09, p = 0.004) and the NG+T2D groups (β = 0.07, p = 0.003; 0.14 p = 0.002) in both the Punjabi and US cohorts, as well as in meta-analysis (β = 0.09, p = 1.58×10^−2^) using either fixed- or random-effect models. This variant also revealed a strong association with total cholesterol in US cohort both in the NG (β = 0.11, p = 0.009) and NG+T2D (β = 0.18, p = 3.58×10^−5^) groups (Table 4).

**Table 5:** Association of significant SNPs with lipid traits in the SDS cohort.

				Punjabi Cohort		US Cohort	
Chr	SNP	Trait	Risk Allele	β (95%CI)	p-value	β (95%CI)	p-value
11	rs964184	TG	G	0.15 (0.09–0.18)	5.94×10^−10^	0.18 (0.09–0.23)	2.46×10^−5^
11	rs12286037	TG	T	0.07 (0.05–0.23)	3.00×10^−3^	0.14 (0.08–0.36)	2.00×10^−3^
16	rs3764261	HDL-C	A	0.09 (0.05–0.14)	1.21×10^−4^	0.10 (0.07–0.13)	1.72×10^−9^
**Primary Meta-analysis (Punjabi and US Cohort)**							
**SNP**	**Trait**	**Risk Allele**	**β** **(Fixed Effect)**	**p-value** **(Fixed Effect)**	**β** **(Random Effect)**	**p-value** **(Random Effect)**	**p-value** **(Heterogeneity)**
rs964184	TG	G	0.16	1.74×10^−17^	0.16	1.74×10^−17^	0.52
rs12286037	TG	T	0.09	1.58×10^−2^	0.09	1.58×10^−2^	0.49
rs3764261	HDL-C	A	0.14	2.03×10^−26^	0.15	4.84×10^−4^	0

### Additional Variants Associated with Serum Lipids

Among other variants, an association for *CELSR2-PSRC1-SORT1* (rs599839) showed a marginally significant decrease in LDL-C (online Table S1). A SNP near *APOE-C1-C4-C2* (rs4420638) showed a moderate association with decreased HDL-C Punjabi cohort and US cohort (online Table S2). Our data could not confirm the association of CDK2A-2B (rs1333049) with lipid traits or T2D (online Table S1, S2).

### Association Analysis of Variants in the LD Region (the chromosomal region 11q23.3) Spanning BUD13-ZNF259, APOA5-A4-C3-A1, and SIK3 Genes with TG

**Figure 1:**
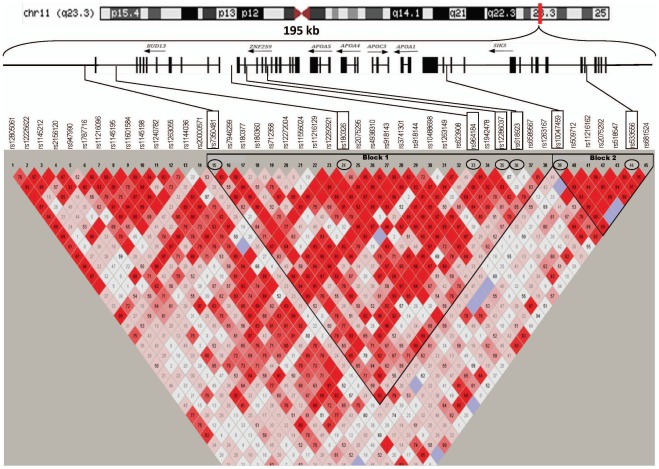
Location of genetic markers in chromosomal region (11q23.3) (195 Kb) encompassing *BUD13-ZNF259, APOA5-A4-C3-A1,* and *SIK3* gene cluster. Exons are shown in black vertical rectangles separated by introns. Significant SNPs (associated with increased triglyceride concentrations) detected in *BUD13*, *ZNF259* and *SIK3* are shown in large rectangles on disequilibrium (LD) matrix with their position on the genes indicated by lines. The direction of transcription of genes is shown in arrows. Pair-wise LD between SNPs (D’) is indicated by diamonds shaded in white-grey-black show the range of LD matrix from D’ = 0 in white to D’ = 1 in black. LD block 1 contains 5 most significant SNPs including two top SNPs (rs964184 and rs7350481) of the total 45 analyzed. LD block 2 shows all SNPs from *the SIK3* gene and the presence of a strong LD among these SNPs containing two strong signals associated with triglycerides in rs10047459 and rs533556.

After seeing strong and consistent association of two variants, rs964184 (*BUD13-ZNF259*) and rs12286037 (*ZNF259*) with TG, we analyzed a further 45 SNPs from the chromosomal region 11q23.3 spanning these two SNPs using genotyping data from our ongoing North Indian (SDS) GWAS and genome-wide data available from 6,530 participants in the London Life Sciences Population (LOLIPOP) study. As shown in Figure 1 and Table 6, six of 45 SNPs revealed a strong association with TG levels in both SDS and LOLIPOP cohorts. Meta-analysis of these variants in the combined sample of 8,530 individuals revealed significant p values in both fixed- and random-effect models. The effect size of each SNP for affecting TG in fixed-effect meta-analysis was (β = 0.20, p = 7.52×10^−26^; β = 0.14, p = 8.15.×10^−21^; β = 0.21, p = 1.06×10^−39^; β = −0.08, p = 3.0×10^−4^; β = 0.08, p = 1.87×10^−8^; β = −0.09, p = 9.28×10^−9^), respectively for rs7350481, rs180326, rs964184, rs618923, rs10047459, rs533556 (Table 6) showing the strongest p value (1.06×10^−39^) for rs964184.

**Table 6:** Association of six most significant SNPs within *BUD13*-*ZNF259*, *A5-A4-C3-A1*, and *SIK3* with TG.

			Punjabi (SDS) Cohort n = 2,000							LOLIPOP Cohort n = 6,530				
SNP	Risk allele	CEU	Allele Frq.		β		SE	p-value		Allele Frq.		β	SE	p-value
rs7350481	A	0.08	0.2		0.12		0.03	9.18×10^−6^		0.21		0.27	0.03	3.91×10^−25^
rs180326	C	0.33	0.35		0.06		0.02	0.009		0.35		0.23	0.02	5.01×10^−26^
rs964184	G	0.12	0.21		0.11		0.02	3.42×10^−6^		0.22		0.36	0.02	3.06×10^−45^
rs618923	A	0.76	0.82		−0.08		0.03	0.002		0.84		−0.07	0.03	0.013
rs10047459	G	0.16	0.38		0.06		0.02	0.007		0.39		0.11	0.02	4.63×10^−7^
rs533556	A	0.3	0.34		−0.06		0.02	0.005		0.36		−0.12	0.02	1.57×10^−7^
		**Joint Meta-analysis** n = 8,530												
		**SNP**	**Risk** **allele**	**β** **(Fixed Effect)**		**p-value** **(Fixed Effect)**			**β** **(Random Effect)**		**p-value** **(Random Effect)**	**p-value** **(Heterogeneity)**		
		rs7350481	A	0.2		7.52×10^−26^			0.19		8.00×10^−3^	2.00×10^−4^		
		rs180326	C	0.14		8.15×10^−21^			0.15		0.09	0		
		rs964184	G	0.21		1.06×10^−39^			0.23		5.80×10^−2^	0		
		rs618923	A	−0.08		3.0×10^−4^			−0.08		2.00×10^−4^	0.851		
		rs10047459	G	0.08		1.87×10^−8^			0.08		7.00×10^−4^	0.094		
		rs533556	A	−0.09		9.28×10^−9^			−0.09		0.001	0.059		

Genotyping on rs12286037 was not available in LOLIPOP sample.

**Figure 2:**
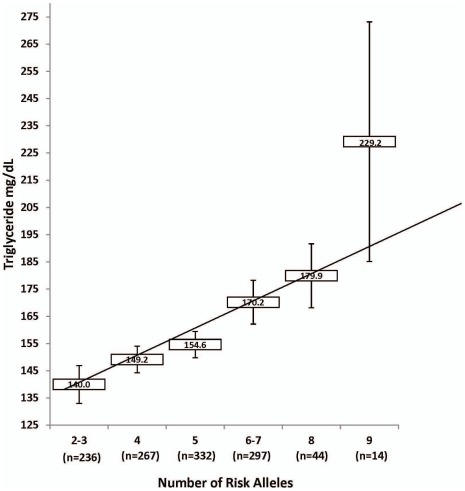
Shows the distribution of serum triglyceride levels in Punjabi, US and entire cohort stratified by rs964184 genotypes. Multiple linear regression analysis was performed using age, BMI and gender in individuals cohorts and age, BMI, gender and place of birth as combined cohorts. *P*-values in the bars show statistically significant association of ‘G’ risk allele with triglycerides.

To further characterize the relationship between genotypes of these variants and their impact on TG levels, we considered the predictive value of the genotype score by counting the number of risk alleles among these seven significant SNPs. As shown in Figure 2, the genotype score of these seven SNPs showed a dose-related increase in TG levels ranging from 140.0±6.9 mg/dL with 2–3 risk alleles to 229.2±44.0 mg/dL with 9 risk alleles. There was an overall increase of 89 mg/dL from 2 to 9 risk alleles (linear regression p = 1.62×10^−6^). Individuals carrying more than 4 risk alleles on average had fasting TG levels greater than the currently acceptable level of TG (150 mg/dL) which would substantially increase their risk for CAD and T2D, and raising implications for early development of complications [16].

Two GWAS SNPs, rs964184 and rs12286037, were in tight LD (D’ = 0.92) with each other in this sample (online Figure S3). We performed step-wise regression to examine the independence of the SNP effects including all significant SNPs along with age, gender, and BMI. Only two SNPs, rs964184 and rs10047459, remained significant in the final model. Interestingly, the strongest signal (β = 0.16, p = 2.57×10^−5^) remained associated with rs964184 for TG (Table 7).

**Table 7:** Test of independence: step-wise multiple linear regression showing association of SNPs with TG using full model‡.

Included in the model			
	Model	Effect	P-value
1	BMI	0.02 (0.01–0.03)	1.33×10^−4^
2	BMI	0.02 (0.01–0.03)	5.25×10^−5^
	rs964184	0.12 (0.05–0.19)	4.73×10^−4^
3	BMI	0.02 (0.01–0.03)	1.15×10^−4^
	rs10047459	0.15 (0.04–0.26)	0.01
	rs964184	0.16 (0.08–0.23)	**2.57×10^−5^**
**Excluded from the model**			
	**Model**	**Effect**	**p-value**
1	Sex	−0.02	0.65
	Age	0.00	0.98
	Disease	0.05	0.26
	rs7350481	0.14	3.05×10^−3^
	rs180326	0.12	0.01
	rs964184	0.16	4.73×10^−4^
	rs12286037	0.10	0.04
	rs10047459	0.07	0.16
	rs533556	−0.11	0.02
	rs618923	−0.07	0.16
2	Sex	−0.03	0.54
	Age	0.00	0.96
	Disease	0.05	0.27
	rs7350481	0.10	0.04
	rs180326	0.09	0.05
	rs12286037	0.05	0.34
	rs618923	−0.13	0.01
	rs10047459	0.13	0.01
	rs533556	−0.08	0.11
3	Sex	−0.04	0.43
	Age	−0.01	0.88
	Disease	0.06	0.22
	rs7350481	0.08	0.10
	rs180326	0.07	0.16
	rs12286037	0.02	0.64
	rs618923	–	–
	rs533556	−0.04	0.48

‡ Includes age, BMI, gender, disease, and rs7350481, rs180326, rs12286037, rs964184, rs618923, rs10047459, rs533556 in the model.

### Haplotype Analysis

To further determine whether SNPs other than rs964184 and rs12286037 account for any additional association with TG when examined together, we performed haplotype analysis using the seven most significant SNPs from the SDS GWAS including rs964184 and rs12286037. As shown in Table 8, the analysis revealed two haplotypes; ACGCAGA carrying ‘G’ risk allele (in rs964184) to be associated with significantly raised TG (β = 0.13, 4.62×10^−6^, empirical p = 9.0×10^−4^), and GACCAAC carrying ‘C’ protective allele to be associated with significant reduced TG concentrations (β = −0.07, p = 0.025, empirical p = 0.034) in this population. The least frequent haplotypes (<5%) were not included in analysis. Note that the association of these haplotypes with TG remained significant (ACGCAGA, p = 2.34×10^−4^ for elevating TG), and (GACCAAC, p = 0.015 for lowering TG) even after controlling for age, gender, and BMI.

**Table 8:** Haplotype association of seven significant SNPs from *BUD13*- *ZNF259*, *A5-A4-C3-A1*, and *SIK3* cluster with TG.

						Controlling for the effect of rs964184		Controlling for the effect of rs12286037		
Haplotype	Frq	β (95% CI)	Unadjusted p	Adjusted p*	Trait effect	β (95% CI)	Adjusted p	β (95% CI)	Adjusted p	Trait effect
ACGCAGA*	0.10	0.13 (0.06 – 0.20)	**4.62×10^−6^**	**2.34×10^−4^**	↑	0.06 (−0.03 – 0.15)	0.204	0.16 (0.09 - 0.23)	**2.83×10^−6^**	↑
GACCAGC	0.06	0.01 (−0.08 – 0.11)	0.826	0.769		0.05 (−0.05 – 0.14)	0.333	0.00 (−0.10 – 0.09)	0.949	
GACCAAA	0.18	−0.02 (0.04 – −0.08)	0.137	0.531		0.01 (−0.05 – 0.07)	0.786	−0.04 (−0.09 – 0.02)	0.221	
GACCAGA	0.13	−0.02 (0.04 – −0.08)	0.350	0.511		−0.01 (−0.07 – 0.05)	0.813	−0.02 (−0.08 – 0.04)	0.434	
GACCAAC^†^	0.18	−0.07 (−0.01 – −0.12)	**0.025**	**0.015**	↓	−0.03 (−0.08 – 0.03)	0.296	−0.05 (−0.11 – 0.00)	**0.047**	↓
GCCCGAC	0.09	−0.08 (−0.01 – −0.16)	0.110	0.034		−0.02 (−0.09 – 0.05)	0.562	−0.05 (−0.13 – 0.02)	0.138	

rs7350481; rs180326; rs964184; rs12286037; rs618923; rs10047459; rs533556; *Empirical p = 9.0×10^−4^; ^†^Empirical p = 0.034; *adjusted for age, gender, and BMI.

To further understand and interpret these findings, we performed conditional haplotype analysis by controlling for the effect of two original SNPs (rs964184 and rs12286037). As shown in the Table 8, the association of ACGCAGA haplotype with increased TG (4.62×10^−6^) and GACCAAC with reduced TG (p = 0.025) levels disappeared after including rs964184 in the model. However, the same haplotypes remained linked with increased TG (ACGCAGA, p = 2.83×10^−6^) and reduced TG (GACCAAC, p = 0.047) levels after controlling for rs12286037. These results further confirm the putative role of rs964184 for independently affecting TG concentrations.

## Discussion

**Figure 3:**
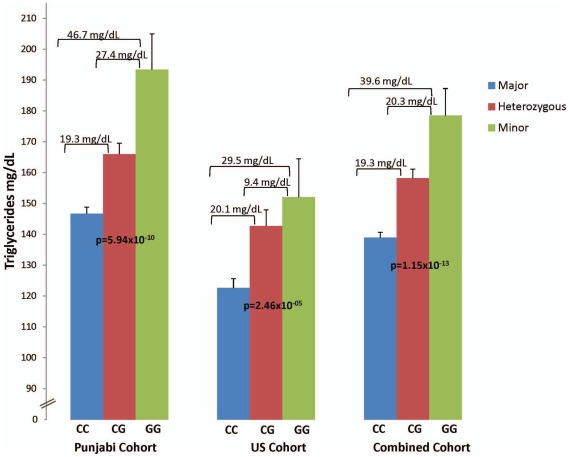
Shows the combined effect of risk alleles of for elevating triglyceride levels from *BUD13* (rs7350481 rs180326), inter-genic variant from *BUD13-ZNF259* (rs964184), and intronic variants from *ZNF259* (12286037 and rs618923), and *SIK3* (rs100447459, rs533556). Y axis represents mean triglyceride levels and X axis represents number of risk alleles with the number of participants per risk allele shown in parenthesis below. Rectangles in the plot indicate mean values of triglycerides separated by each risk-allele group and error bars are 95% CI. Note that the the cumulative gene-score of all significant SNPs showed a dose-related increase in TG concentrations ranging from (140.0±6.9 mg/dL with 2–3 risk alleles to 229.2±44.0 mg/dL with 9 risk allele carriers with overall effect increased to 89 mg/dL from 2 to 9 risk alleles (p = 1.62×10^−6^ ).

Our study has convincingly replicated the associations of two of the six most associated GWAS SNPs with blood lipid phenotypes in a non-European population. We previously reported a strong association of rs3764261 from the promoter region of *CETP* gene with HDL-C in our Punjabi cohort (n = 2,431) [17]. Our current data also provide strong evidence of association of rs3764261 with HDL-C in our expanded cohort (Punjabi+US) separately (Punjabi: n = 2,902, β = 0.09, 6.31×10^−5^; US Asian Indians: n = 879, β = 0.10, 1.72×10^−9^), and combined in a meta-analysis (n = 3,781, β = 0.14, 2.03×10^−26^). The serum HDL-C levels increased 13% in ‘AA’ carriers over those of common ‘CC’ carriers. These results are in agreement with this ‘A’ allele being associated with raised HDL-C levels reported in previous GWAS and meta-analysis studies in Caucasians [13], [18]. The other important confirmation in our findings was the robust association of TG concentrations in this cohort with rs964184 from the inter-genic region between *BUD13 and ZNF259,* and rs12286037 an intronic variant from *ZNF259* near *APOA5-A4-C3-A1*. The *APOA5-A4-C3-A1* locus is associated with plasma TG and VLDL-C levels in several studies including Caucasian GWAS and meta-analyses [8], [18], Chinese [19], Asian Indians from UK [20], US Whites and Blacks [21], and Middle-Easterns [22]. Notably, in our study, the allelic effects of these variants were stronger under conditions of dyslipidemia associated with T2D and the difference in effect size (β = 0.16 T2D vs. β = 0.10 NG control) for rs964184 was statistically significant (p = 0.01). These results agree with earlier studies where the effect size of the loci contributing to quantitative traits of CAD was magnified under conditions of diabetes [23], [24]. It also was interesting to observe that not only the same risk alleles, ‘G’ of rs964184 (*BUD13*-*ZNF259*) and ‘T’ of rs12286037 (*ZNF259*) were involved in raising TG levels but also the effect sizes for per ‘G’ allele increase in TG was also similar in our sample (19.3 mg/dL Punjabi), (20.1 mg/dL US) and (19.3 mg/dL pooled) (Figure 3) when compared to European populations (18.12 mg/dL) [18]. After further exploration of this region 11q23.3 using 45 SNPs from this locus, other SNPs in LD with the lead SNP (rs964184) were also associated with TG showing high significance in the SDS and LOLIPOP cohorts individually and in meta-analysis (Table 6). In the presence of LD across the region, the precise causal variant remains to be identified.

Upon analyzing these variants together in haplotype analysis, two frequent haplotypes- ACGCAGA (frequency 10%) and GACCAAC (frequency 18%) revealed a strongly significant association with TG concentrations. The major effect appears to be driven by rs964184 as the association of this haplotype (ACGCAGA) with TG was no longer significant after analyzing this haplotype combination conditional upon rs964184 (β = 0.06, p = 0.204). However, the same haplotype (ACGCAGA) showed strong association with raised TG levels (β = 0.16, p = 2.83×10^−6^) when analysis was controlled for rs12286037 (Table 8).

Our data show a weak association of rs599839, representing *CELSR2-PSRC1-SORT1,* with reduced LDL-C levels in the Punjabi cohort (β = −0.06, p = 0.011) and a non-significant trend in the US cohort (β = −0.03, p = 0. 572) (online Tables S1 and S2). This same variant was associated with LDL-C in Chinese (p<0.001), Asian Indians (p = 0.003), and Malays (p = 0.004) from Singapore [8] and showed a strong association with LDL-C in a large-scale replication study in Japanese (p = 3.1×10^−11^) [25]. Our study could not replicate the association of the remaining variants, especially the *APOE-CI-C4-C2* cluster variant rs4420638 with LDL-C as reported in a Caucasian GWAS [26], and meta-analysis [7]. Instead, our data showed a similar minor (at risk) allele-associated decrease in HDL-C in both the Punjabi (β = −0.06, p = 0.007) and US (β = −0.09, p = 0.032) cohorts. Our data did not confirm associations of *CDKN2A-2B* (rs1333049) with T2D, CAD, FBG, fasting insulin, or lipids as reported in earlier studies [27]. We previously reported negative association of another variant in *CDKN2A-2B* (rs10811661) with T2D and other-related traits in this population [15] contrary to associations seen in Caucasian populations [28], [29]. The negative association of these loci could be due to population stratification, phenotype heterogeneity, evolutionary pressures, demographic and cultural histories or a lack of power in our study to detect these small effects as significant. Perhaps gene x gene interactions and gene x environment interactions, or phenotypic variability due to differences in biological adaptation or other factors are the cause for the poor replication [11]. Many times the high risk variant may be restricted to certain populations, for instance, the restricted association of *KCNQ1* SNPs (rs2237892, rs2237897) with T2D in East Asians because of the significant variation of allele frequency across ethnic groups [30]. On the other hand, if the same variant is showing association with disease or traits in diverse populations, validation studies enable more generalizable estimates of effect sizes in the general population [31].

It is interesting to observe that the variants identified by GWAS, especially those related to lipid regulation also are associated with CAD. A CAD risk locus associated with rs599839 in the *CELSR2-PSRC1-SORT1* region was not only associated with elevated LDL-C concentrations, but also with CAD [32]. These findings suggest that the locus association with CAD may be mediated though its effect on LDL-C levels, although we could not confirm the role of this variant (rs599839) with CAD in this sample. On the other hand, many times the relationship of a SNP with a trait may be direct but not with the main disease due to the multifactorial nature of the disease. For instance, within the 11q23.3 region, although our findings revealed a direct causal relationship between the SNP and the trait (TG), none of the variants from this locus was associated with T2D or CAD as has been observed for the LDL-CAD locus on chromosome 1. The ‘less common’ variants possibly reveal a ‘common’ association with TG and disease (T2D/CAD). A recent targeted resequencing study conducted on patients with severe hypertriglyceridemia (HTG) for *APOA5* detected an abundance of rare variants in HTG patients with T2D in comparison to those without T2D (25% vs. 6.1%, p = 0.037) [33]. These findings suggest the co-inheritance of TG raising alleles with other physiological factors operating together in the common pathway leading to T2D. Even in this investigation, the allelic contribution of the SNP rs964184 was increased from β = 0.10 in non-diabetics to β = 0.16 in diabetics (p = 0.01) (Table 3).

Most of these GWAS variants belong to inter-genic or non-coding regions. These may have influence on the transcriptional binding sites of the adjacent genes or may interfere with the transcriptional mechanisms without being directly involved in protein regulation. The *ZNF259* gene is located ∼1.6 Kb upstream of the *APOA5-A4-C3-A1* gene cluster, and the top ranking SNP influencing TG levels (rs964184) resides in the intergenic region between *BUD-13* and *ZNF259. ZNF259* is a regulatory protein involved in cell proliferation and signal transduction and may have multiple physiological functions [34]. The most relevant transcription factors that bind to the promoter site of *ZNF259* include proxisome proliferator activated receptor gamma *(PPARG1 and PPARG2)*, and hepatocyte nuclear receptor alpha (*HNF4α1 and HNF4α2*). Nuclear receptors *PPARG* 1 and 2 are expressed in diverse tissues and have been used as targets for improving insulin sensitivity and are widely studied for their role in insulin sensitivity and obesity together with influencing the transcription of several target genes [35], [36]. *HNF4α 1* and *2* nuclear receptors are linked to several human diseases and are known to activate a variety of genes involved in glucose, fatty acid, and cholesterol metabolism in the liver, kidney, intestine, and pancreas [37]. Therefore, an in-depth study of the remotely controlled regulatory mechanisms is needed to clarify which SNPs are functional and how these genes actually influence circulating TG concentrations.

Although none of the six SNPs most associated with TG actually belong to the *APOA5-A4-C3-A1* gene cluster the presence of two top signals (rs964184, p = 1.06×10^−39^ and rs7350481, p = 7.52×10^−26^) within this LD region (stretching up to ∼65.9 Kb interval in block 1) (Figure 1 and Table 6) suggests the possible presence of rare or less frequent causal variants in this region. Confirmation of positive associations in some of the strongest GWAS signals, *CETP* (rs3726461) with HDL-C and *BUD13-ZNF259* (rs964184) with TG, in these independently ascertained non-European populations of Indian origin validate the strength of GWAS studies and their usefulness and potential to find disease loci affecting complex chronic disorders. However, the identified genes and inter-genic variants most likely represent just the tip of the iceberg for cardiovascular risk as the overall residual variance contributed by these SNPs is <5% and even the meta-analysis ORs do not exceed 1.22. These findings suggest that rarer or less common variants which are currently invisible in GWAS may exist within these regions. Further fine mapping and targeted resequencing in these gene regions in different ethnicities, as well as functional studies, would help detection of putative loci of therapeutic significance.

## Methods

### Human Subjects- Punjabi and US Cohorts

DNA and serum samples from a total of 3,781 individuals (2,902 Punjabi Cohort [52% T2D]; 879 US Cohort [16%T2D]) were studied. The healthy control participants from the Punjabi cohort were random unrelated individuals recruited from the same Asian Indian community as the T2D patients and matched for ethnicity and geographic location. The US subjects were recruited through public advertisement as part of a population-based study involving free health screening for cardiovascular risk factors. The individuals with mixed ancestry or non-Asian Indian ancestry were not enrolled. Two third of the participants from the US cohort were originally from the state of Punjab, and the remaining one third were from other western and southern states of India. Men and women aged 25–79 years participated. The diagnoses of T2D were confirmed by reviewing medical records for symptoms, use of medication, and measuring FBG levels following the guidelines of the American Diabetes Association (2004) [38], as described in detail previously [39]. A medical record indicating either (1) a FBG *≥*126 mg/dL or *≥*7.0 mmol/L after a minimum 12 h fast or (2) a 2 h post-glucose level (2 h oral glucose tolerance test) *≥*200 mg/dL or *≥*11.1 mmol/L on more than one occasion, combined with symptoms of diabetes, confirmed the diagnosis. Impaired fasting glucose (IFG) was defined as a fasting blood glucose level *≥*100 mg/dL (5.6 mmol/L) but *≤*126 mg/dL (7.0 mmol/L). Impaired glucose tolerance (IGT) was defined as a 2 h OGTT >140 mg/dL (7.8 mmol/L) but <200 mg/dL (11.1 mmol/L). Participants with IFG or IGT were considered pre-diabetics and were analyzed separately. The 2h OGTTs were performed following the criteria of the World Health Organizations (WHO) (75 g oral load of glucose). BMI was calculated as (weight [kg]/height [meter]^2^). Participants with type I diabetes, or those having a family member with type I diabetes, or rare forms of T2D sub-types (maturity onset diabetes of young [MODYs]), or secondary diabetes (from e.g. hemochromatosis, pancreatitis) were excluded from the study.

Controls, clinically free of T2D, IGT, or IFG, were selected based on a fasting glycemia <100.8 mg/dL (<5.6 mmol/L) or a 2 h glucose <141.0 mg/dL (<7.8 mmol/L). Participants with IFG or IGT were excluded when data were analyzed for association of variants with T2D. All blood samples were obtained at the baseline visits. All participants signed a written informed consent for the investigations. The study was reviewed and approved by the University of Oklahoma Health Sciences Center’s Institutional Review Board, as well as the Human Subject Protection Committees at the participating hospitals and institutes in India.

### Metabolic Assays

Insulin was measured by radio-immuno assay (Diagnostic Products, Cypress, USA). HOMA IR (fasting glucose x fasting insulin)/22.5 and HOMA B (fasting insulin x 20/FBG −3.5), were calculated as described [40]. Serum lipids [total cholesterol, LDL-C, HDL-C, VLDL-C, and TG] were measured using standard enzymatic methods (Roche, Basel, Switzerland) as described previously [41].

### SNP Genotyping

We genotyped six SNPs from GWAS derived loci (*CELSR2-PSRC1-SORT1* rs599839; *CDKN2A-2B* rs1333049; *BUD13*-*ZNF259* rs964184; *ZNF259* rs12286037; *CETP* rs3764261; *APOE-C1-C4-C2* rs4420638). Details of the investigated loci, their previously reported association with lipid phenotypes (traits), allele frequency, effect size, population studied etc. are summarized in Table 2. Genotyping for these six SNPs was performed using TaqMan pre-designed or TaqMan made-to-order SNP genotyping assays from Applied Biosystems Inc. (ABI, Foster City, USA). Genotyping reactions were performed on an ABI 7900HT genetic analyzer using 2 uL of genomic DNA (10 ng/uL), following manufacturers’ instructions. For quality control, 8–10% replicate controls and 4–8 negative controls were used in each 384 well plate to match the concordance, and the discrepancy rate in duplicate genotyping was <0.2%. Genotyping call rate was 97% or more in all the SNPs studied.

### LOLIPOP Cohort (UK)

Assessment of LOLIPOP participants was carried out by trained research nurses, according to a standardized protocol and with regular quality control (QC) audits as described previously [42]. T2D cases were selected based on physician diagnosis of diabetes on treatment, with onset of diabetes after the age of 18 years and without insulin use in the first year after diagnosis, or FBG >126 mg/dL on 2 or more occasions [38]. Controls were selected based on no history of diabetes, and FBG <110 mg/dL. An interviewer-administered questionnaire was used to collect data on medical history, family history, current prescribed medication (verified from the practice computerized records), cardiovascular risk factors, alcohol intake, physical activity, and socio-economic status. Country of birth of participants, parents, and grandparents was recorded together with language and religion for assignment of ethnic subgroups. Physical assessments including blood pressure, anthropometric measurements (height, weight, and WHR), fat mass (bio-impedance), urinalysis, and 12 lead ECG. FBG, insulin, total, HDL-C and LDL-C, TG, were measured on all participants as described previously [6]. At the time of this analysis genotype and phenotype data on 6,530 individuals comprising 1,774 T2D cases and 4,756 controls were available from this study.

### GWAS

Genome-wide association scans in LOLIPOP and SDS samples were performed using Illumina Infinium Beadchips genotypes were called using GenCall or Illuminus algorithms. Samples with a SNP call rate <95% were removed, as were SNPs with call rate <97%, minor allele frequency <1%, or HWE p<1.0×10^−6^. Principal components analysis (PCA) was used in both GWAS datasets to control for population stratification by comparison to reference samples from the Hapmap YRI, CHB, JPT and CEU panels using PLINK (http://pngu.mgh.harvard.edu/~purcell/plink/) and Eigensoft [43], and the Indian samples collected by Reich and colleagues [44]. Samples with eigenvalues inconsistent with Asian Indian ancestry were removed as described previously [45].

### Statistical Analysis

Data quality for SNP genotyping was checked by establishing reproducibility of control DNA samples. Departure from HWE in controls was tested using the Pearson chi-square test. The genotype and allele frequencies in T2D cases were compared to those in control subjects using the chi-square test. Statistical evaluation of genetic effects on T2D risk used multivariate logistic regression analysis with adjustments for age, gender, and other covariates. Continuous traits with skewed sampling distributions (e.g., TG and total cholesterol) were log-transformed before statistical analysis. However, for illustrative purposes, values were re-transformed into the original measurement scale. Supplementary Figure S2 shows the distribution of serum TG levels before and after transformation. General linear models were used to test the impact of genetic variants on transformed continuous traits. Country of birth was used as a covariate when analyzing the combined sample of the Punjabi and US cohorts. Other significant covariates for each dependent trait were identified by Spearman’s correlation and step-wise multiple linear regression with an overall 5% level of significance using SPSS for Windows statistical package (version 18.0) (SPSS Inc., Chicago, USA). Mean values between cases and controls were compared by using an unpaired t-test. To adjust for multiple testing, we used Bonferroni’s correction (0.05/number of tests performed).

Haplotype analysis of *BUD13-ZNF259* rs964184, *ZNF259* rs12286037, and other significant SNPs analyzed from the 195 Kb region surrounding these two variants was performed using HAPLOVIEW (version 4.0) which uses an accelerated expectation maximization algorithm to calculate haplotype frequencies (http://www.broadinstitute.org/haploview/haploview). Effect of seven-site haplotype on quantitative traits were determined using PLINK. Meta-analysis was performed by using PLINK for fixed-effects and random-effects models and the p value for heterogeneity was derived from Cochrane’s Q statistics. The fixed effect meta-analysis is based on the assumption that a single common (or fixed) effect underlies each study in the meta-analysis. Random effect meta-analysis provides information about the distribution of effects across different studies. Design of the meta-analysis is described in a flow chart (online Figure S1).

Statistical power was assessed using the Genetic Power Calculator [46]. The general estimates of power in the Punjabi and combined sample using an additive genetic model at α = 0.05, K = 0.18 for detecting the effect sizes between 1.12 and 1.58 for T2D, were 56% and 89% in the Punjabi and 66% and 97% in combined cohorts, respectively, when the frequency of risk alleles were 0.82 and 0.35, respectively, in our sample. However, for quantitative traits, the power was well in excess (90%) to detect the inter-genotype difference (e.g. for TG levels), assuming an additive genetic model, (α = 0.05, and Bonferroni’s p = 0.008) at allele frequencies ranging from 0.05–0.89 using, 1,262, 569, and 1,861 controls from the Punjabi, US, and combined cohorts, respectively. This power is associated to detect a difference in a quantitative trait of TG of as little as 1 mg/dL and accounts for an effect size of 0.1 which corresponds to detecting significant β's outside of the range of ±0.05.

## Supporting Information

Figure S1 **Flowchart showing step-wise plan and inclusion of studies in meta-analysis.**
(TIFF)

Figure S2 **Histogram plots showing distribution of serum triglycerides and HDL cholesterol before and after log transformation.**
(TIFF)

Figure S3 **Linkage disequilibrium between two GWAS SNPs (rs964184 and rs12286037) association with serum triglycerides.**
(TIFF)

Table S1 **Association of SNPs with lipid traits in Punjabi cohort.**
(DOCX)

Table S2 **Association of SNPs with lipid traits in US cohort.**
(DOCX)
