# Supplementary figures and images for: A Replication Study of GWAS-Derived Lipid Genes in Asian Indians: The Chromosomal Region 11q23.3 Harbors Loci Contributing to Triglycerides
Source: PLoS One. 2012 May 18;7(5):e37056. doi: 10.1371/journal.pone.0037056 (PMC3356398; doi:10.1371/journal.pone.0037056)

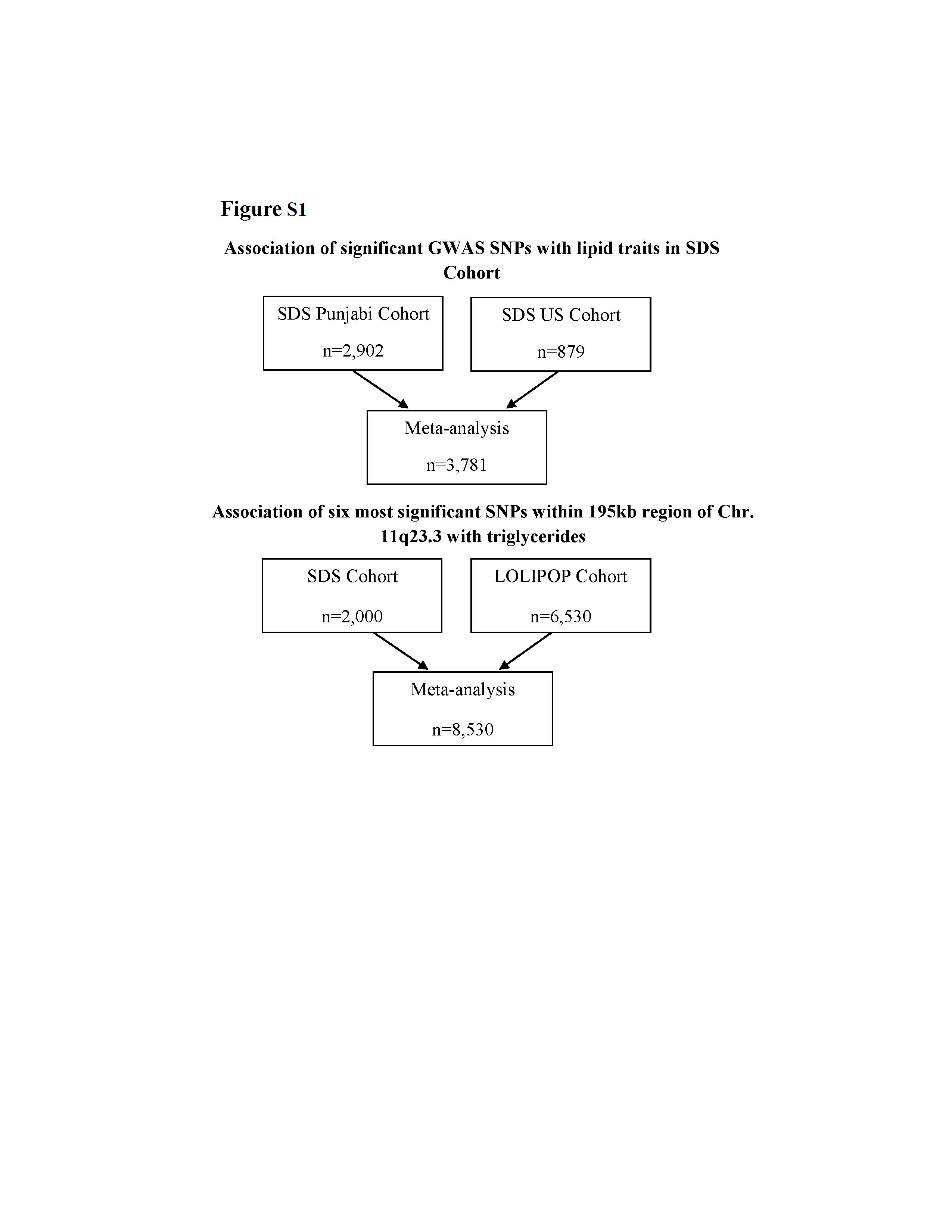

Supplement: Figure S1 — Flowchart showing step-wise plan and inclusion of studies in meta-analysis. (TIFF) [file pone.0037056.s001.tiff]

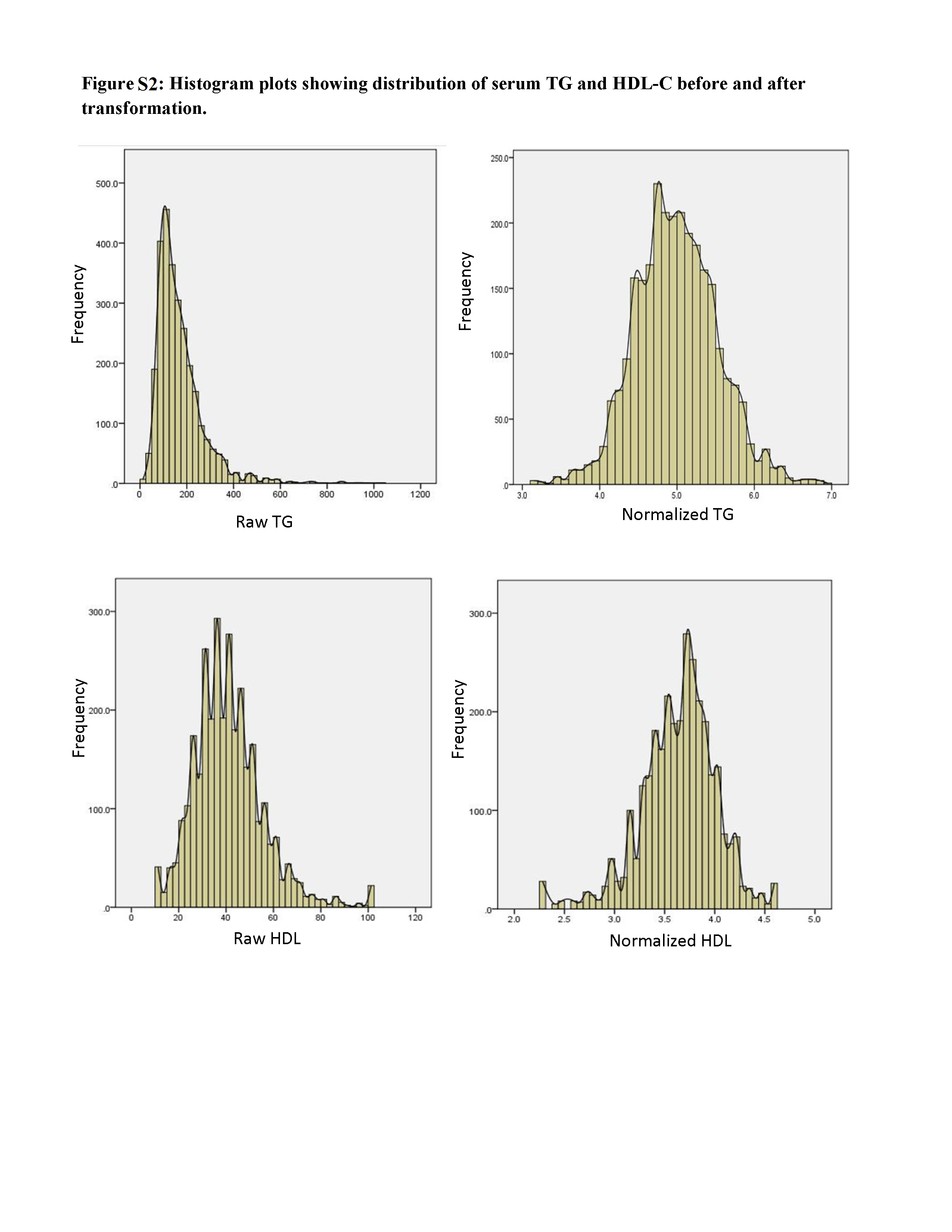

Supplement: Figure S2 — Histogram plots showing distribution of serum triglycerides and HDL cholesterol before and after log transformation. (TIFF) [file pone.0037056.s002.tiff]

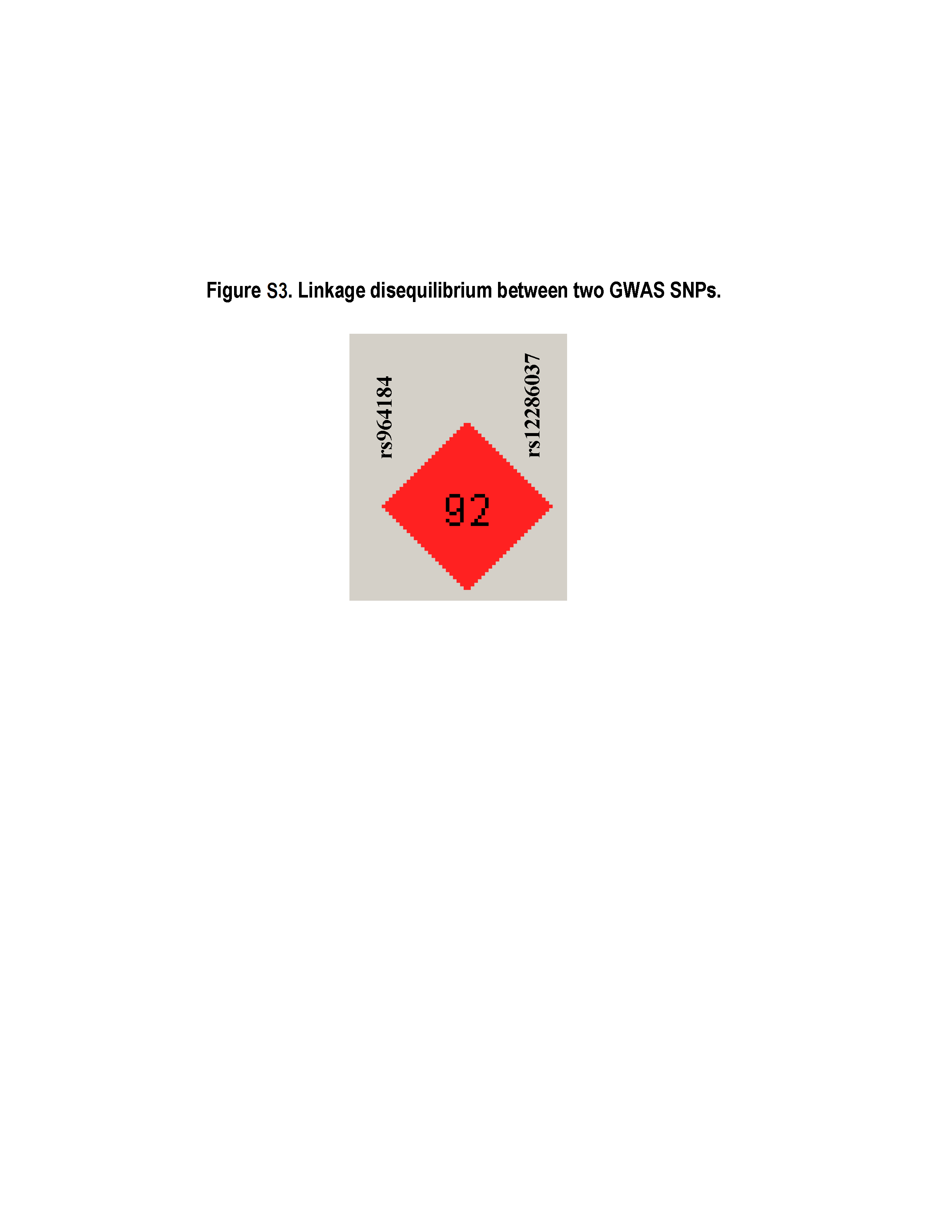

Supplement: Figure S3 — Linkage disequilibrium between two GWAS SNPs (rs964184 and rs12286037) association with serum triglycerides. (TIFF) [file pone.0037056.s003.tiff]
