# Supplementary material for: A Replication Study of GWAS-Derived Lipid Genes in Asian Indians: The Chromosomal Region 11q23.3 Harbors Loci Contributing to Triglycerides
Source: PLoS One. 2012 May 18;7(5):e37056. doi: 10.1371/journal.pone.0037056 (PMC3356398; doi:10.1371/journal.pone.0037056)
Supplement: Table S1 — Association of SNPs with lipid traits in Punjabi cohort. (DOCX) [file pone.0037056.s004.docx]

| **Table S1. Association of SNPs with lipid traits in Punjabi Cohort** | | | | | | | | | | | | | | | | | | |
| --- | --- | --- | --- | --- | --- | --- | --- | --- | --- | --- | --- | --- | --- | --- | --- | --- | --- | --- |
| **NG Controls** | | | | | | | **T2D Cases** | | | | | | **Combined (NG Controls + T2D Cases)** | | | | | |
|  | **β** | **p-value** | β | **p-value** | **β** | **p-value** | **β** | **p-value** | **β** | **p-value** | **β** | **p-value** | **β** | **p-value** | **β** | **p-value** | **β** | **p-value** |
| ***CELSR2-PSRC1-SORT1* rs599839** | log additive | | dominant | | recessive | | log additive | | dominant | | recessive | | log additive | | dominant | | recessive | |
| Cholesterol (mg/dL) | -0.02 | 0.648 | -0.01 | 0.807 | -0.02 | 0.528 | -0.06 | 0.038 | -0.05 | 0.105 | -0.06 | 0.060 | -0.05 | 0.066 | -0.04 | 0.120 | -0.04 | 0.144 |
| TG (mg/dL) | -0.02 | 0.483 | -0.02 | 0.673 | -0.03 | 0.379 | -0.02 | 0.542 | -0.02 | 0.542 | -0.01 | 0.748 | -0.02 | 0.414 | -0.02 | 0.474 | -0.02 | 0.546 |
| HDL-C (mg/dL) | 0.06 | 0.066 | 0.06 | 0.086 | 0.04 | 0.246 | -0.01 | 0.789 | -0.01 | 0.708 | 0.00 | 0.963 | 0.02 | 0.508 | 0.01 | 0.688 | 0.02 | 0.492 |
| LDL-C (mg/dL) | -0.04 | 0.239 | -0.04 | 0.285 | -0.03 | 0.426 | -0.06 | **0.027** | -0.05 | 0.104 | -0.06 | **0.029** | -0.06 | **0.011** | -0.05 | **0.042** | -0.05 | **0.025** |
|  |  | |  | |  | |  | |  | |  | |  | |  | |  | |
| ***CDKN2A-2B* rs1333049** | log additive | | dominant | | recessive | | log additive | | dominant | | recessive | | log additive | | dominant | | recessive | |
| Cholesterol (mg/dL) | 0.00 | 0.902 | 0.04 | 0.209 | -0.05 | 0.134 | 0.01 | 0.835 | 0.01 | 0.772 | 0.00 | 0.959 | -0.01 | 0.603 | 0.00 | 0.889 | -0.02 | 0.318 |
| TG (mg/dL) | -0.01 | 0.808 | 0.02 | 0.489 | -0.04 | 0.263 | 0.01 | 0.787 | 0.03 | 0.409 | -0.01 | 0.704 | 0.01 | 0.699 | 0.03 | 0.241 | -0.01 | 0.586 |
| HDL-C (mg/dL) | -0.06 | 0.077 | -0.04 | 0.261 | -0.06 | 0.071 | -0.04 | 0.202 | -0.05 | 0.079 | -0.01 | 0.736 | -0.06 | **0.005** | -0.06 | **0.007** | -0.04 | 0.060 |
| LDL-C (mg/dL) | 0.04 | 0.226 | 0.05 | **0.048** | 0.00 | 0.969 | -0.04 | 0.206 | -0.02 | 0.448 | -0.04 | 0.189 | 0.00 | 0.911 | 0.01 | 0.681 | -0.01 | 0.543 |
|  |  | |  | |  | |  | |  | |  | |  | |  | |  | |
| ***BUD13-ZNF259* rs964184** | log additive | | dominant | | recessive | | log additive | | dominant | | recessive | | log additive | | dominant | | recessive | |
| Cholesterol (mg/dL) | 0.05 | 0.118 | 0.03 | 0.370 | 0.11 | **0.024** | 0.02 | 0.473 | 0.01 | 0.658 | 0.03 | 0.356 | 0.04 | 0.122 | 0.03 | 0.240 | 0.04 | 0.121 |
| TG (mg/dL) | 0.10 | **0.001** | 0.11 | **0.003** | 0.20 | **0.011** | 0.16 | **9.63x10^-7^** | 0.13 | **3.09x10^-5^** | 0.13 | **6.94x10^-5^** | 0.15 | **5.94x10^-10^** | 0.14 | **3.52x10^-8^** | 0.11 | **6.01x10^-6^** |
| HDL-C (mg/dL) | 0.01 | 0.769 | -0.02 | 0.610 | 0.06 | 0.052 | -0.03 | 0.321 | -0.03 | 0.261 | -0.01 | 0.820 | 0.00 | 0.961 | -0.01 | 0.770 | 0.02 | 0.446 |
| LDL-C (mg/dL) | 0.01 | 0.793 | 0.00 | 0.918 | 0.02 | 0.632 | -0.04 | 0.170 | -0.04 | 0.204 | -0.03 | 0.371 | -0.02 | 0.418 | -0.02 | 0.504 | -0.02 | 0.485 |
|  |  | |  | |  | |  | |  | |  | |  | |  | |  | |
| ***ZNF259* rs12286037** | log additive | | dominant | | recessive | | log additive | | dominant | | recessive | | log additive | | dominant | | recessive | |
| Cholesterol (mg/dL) | 0.01 | 0.742 | 0.01 | 0.876 | -- | -- | -0.01 | 0.860 | -0.02 | 0.625 | 0.06 | 0.052 | -0.02 | 0.361 | -0.03 | 0.232 | 0.05 | **0.045** |
| TG (mg/dL) | 0.02 | 0.487 | 0.02 | 0.534 | -- | -- | 0.09 | **0.004** | 0.09 | **0.005** | 0.03 | 0.326 | 0.07 | **0.003** | 0.07 | **0.003** | 0.02 | 0.331 |
| HDL-C (mg/dL) | -0.01 | 0.867 | -0.01 | 0.686 | -- | -- | -0.04 | 0.200 | -0.05 | 0.111 | 0.04 | 0.221 | -0.03 | 0.129 | -0.04 | 0.076 | 0.03 | 0.214 |
| LDL-C (mg/dL) | -0.01 | 0.802 | -0.01 | 0.683 | -- | -- | -0.03 | 0.348 | -0.03 | 0.302 | 0.01 | 0.790 | -0.03 | 0.179 | -0.03 | 0.135 | 0.01 | 0.533 |
|  |  | |  | |  | |  | |  | |  | |  | |  | |  | |
| ***CETP* rs3764261** | log additive | | dominant | | recessive | | log additive | | dominant | | recessive | | log additive | | dominant | | recessive | |
| Cholesterol (mg/dL) | 0.03 | **0.034** | 0.06 | 0.093 | 0.06 | 0.064 | -0.03 | 0.291 | 0.00 | 0.935 | -0.06 | 0.056 | 0.02 | 0.406 | 0.02 | 0.334 | 0.01 | 0.765 |
| TG (mg/dL) | -0.02 | 0.546 | -0.02 | 0.594 | -0.02 | 0.654 | -0.12 | **1.02x10^-4^** | -0.12 | **1.90x10^-4^** | -0.08 | **0.013** | -0.08 | **0.002** | -0.08 | **0.002** | -0.04 | 0.080 |
| HDL-C (mg/dL) | 0.09 | **1.14x10^-6^** | 0.10 | **1.32x10^-4^** | 0.15 | **6.71x10^-5^** | 0.07 | **0.014** | 0.04 | 0.136 | 0.08 | 0.007 | 0.09 | **1.21x10^-4^** | 0.06 | **0.011** | 0.09 | **6.31x10^-5^** |
| LDL-C (mg/dL) | 0.01 | 0.672 | 0.02 | 0.621 | 0.01 | 0.887 | 0.01 | 0.867 | 0.02 | 0.464 | -0.02 | 0.513 | 0.02 | 0.470 | 0.03 | 0.230 | -0.01 | 0.815 |
|  |  | |  | |  | |  | |  | |  | |  | |  | |  | |
| ***APOE-C1-C4-C2* rs4420638** | log additive | | dominant | | recessive | | log additive | | dominant | | recessive | | log additive | | dominant | | recessive | |
| Cholesterol (mg/dL) | -0.03 | 0.330 | -0.02 | 0.573 | -0.23 | **0.040** | -0.04 | 0.234 | -0.04 | 0.158 | 0.04 | 0.160 | -0.02 | 0.541 | -0.02 | 0.458 | 0.02 | 0.503 |
| TG (mg/dL) | 0.00 | 0.962 | 0.00 | 0.992 | 0.01 | 0.787 | 0.02 | 0.501 | 0.02 | 0.532 | 0.02 | 0.639 | 0.02 | 0.380 | 0.02 | 0.450 | 0.02 | 0.354 |
| HDL-C (mg/dL) | -0.06 | 0.053 | -0.05 | 0.114 | -0.34 | **0.027** | -0.07 | **0.016** | -0.07 | **0.017** | -0.02 | 0.496 | -0.06 | **0.007** | -0.06 | **0.012** | -0.04 | 0.064 |
| LDL-C (mg/dL) | 0.02 | 0.566 | 0.02 | 0.522 | -0.01 | 0.821 | 0.01 | 0.862 | 0.00 | 0.982 | 0.04 | 0.174 | 0.01 | 0.545 | 0.01 | 0.603 | 0.02 | 0.498 |
|  | | | | | | | | | | | | | | | | | | |
