# Supplementary material for: A Replication Study of GWAS-Derived Lipid Genes in Asian Indians: The Chromosomal Region 11q23.3 Harbors Loci Contributing to Triglycerides
Source: PLoS One. 2012 May 18;7(5):e37056. doi: 10.1371/journal.pone.0037056 (PMC3356398; doi:10.1371/journal.pone.0037056)
Supplement: Table S2 — Association of SNPs with lipid traits in US cohort. (DOCX) [file pone.0037056.s005.docx]

| **Table S2. Association of SNPs with lipid traits in US Cohort** | | | | | | | | | | | | |
| --- | --- | --- | --- | --- | --- | --- | --- | --- | --- | --- | --- | --- |
| **NG Controls** | | | | | | | **Combined (NG Controls + T2D Cases)** | | | | | |
|  | **β** | **p-value** | **β** | **p-value** | **β** | **p-value** | **β** | **p-value** | **β** | **p-value** | **β** | **p-value** |
| ***CELSR2-PSRC1-SORT1* rs599839** | log additive | | dominant | | recessive | | log additive | | dominant | | recessive | |
| Cholesterol (mg/dL) | -0.05 | 0.243 | -0.03 | 0.492 | -0.07 | 0.129 | -0.01 | 0.446 | -0.01 | 0.760 | -0.04 | 0.197 |
| TG (mg/dL) | -0.02 | 0.596 | -0.04 | 0.351 | 0.02 | 0.616 | -0.01 | 0.829 | -0.03 | 0.511 | 0.04 | 0.420 |
| HDL-C (mg/dL) | -0.02 | 0.701 | -0.03 | 0.518 | 0.01 | 0.759 | -0.01 | 0.899 | 0.01 | 0.852 | -0.03 | 0.479 |
| LDL-C (mg/dL) | -0.02 | 0.368 | -0.02 | 0.408 | -0.03 | 0.558 | -0.03 | 0.572 | -0.02 | 0.704 | -0.03 | 0.514 |
|  |  | |  | |  | |  | |  | |  | |
| ***CDKN2A-2B* rs1333049** | log additive | | dominant | | recessive | | log additive | | dominant | | recessive | |
| Cholesterol (mg/dL) | -0.03 | 0.554 | -0.01 | 0.871 | -0.03 | 0.427 | 0.01 | 0.669 | 0.01 | 0.814 | 0.01 | 0.643 |
| TG (mg/dL) | -0.01 | 0.789 | -0.01 | 0.837 | -0.01 | 0.816 | -0.01 | 0.903 | -0.03 | 0.485 | 0.02 | 0.627 |
| HDL-C (mg/dL) | -0.01 | 0.810 | -0.01 | 0.876 | -0.01 | 0.814 | -0.05 | 0.191 | -0.02 | 0.575 | -0.06 | 0.116 |
| LDL-C (mg/dL) | -0.01 | 0.644 | -0.03 | 0.389 | 0.00 | 0.927 | -0.02 | 0.667 | -0.03 | 0.443 | 0.00 | 0.951 |
|  |  | |  | |  | |  | |  | |  | |
| ***BUD13-ZNF259* rs964184** | log additive | | dominant | | recessive | | log additive | | dominant | | recessive | |
| Cholesterol (mg/dL) | 0.08 | 0.059 | 0.07 | 0.101 | 0.06 | 0.147 | 0.08 | 0.065 | 0.09 | 0.049 | 0.03 | 0.459 |
| TG (mg/dL) | 0.12 | **0.005** | 0.13 | **0.002** | **0.03** | 0.484 | 0.18 | **2.46x10^-5^** | 0.19 | **1.12x10^-5^** | 0.08 | 0.058 |
| HDL-C (mg/dL) | -0.10 | **0.012** | -0.10 | **0.021** | **-0.09** | **0.037** | -0.08 | 0.057 | -0.07 | 0.073 | -0.05 | 0.224 |
| LDL-C (mg/dL) | 0.03 | 0.116 | 0.03 | 0.201 | 0.08 | 0.161 | 0.02 | 0.216 | 0.03 | 0.539 | 0.07 | 0.116 |
|  |  | |  | |  | |  | |  | |  | |
| ***ZNF259* rs12286037** | log additive | | dominant | | recessive | | log additive | | dominant | | recessive | |
| Cholesterol (mg/dL) | 0.11 | **0.009** | 0.11 | **0.014** | 0.06 | 0.140 | 0.18 | **3.58x10^-5^** | 0.17 | **1.09x10^-4^** | 0.10 | **0.030** |
| TG (mg/dL) | 0.07 | 0.087 | 0.07 | 0.102 | 0.04 | 0.374 | 0.14 | **0.002** | 0.13 | **0.002** | 0.06 | 0.162 |
| HDL-C (mg/dL) | -0.01 | 0.740 | -0.01 | 0.835 | -0.03 | 0.528 | -0.01 | 0.808 | -0.02 | 0.717 | 0.02 | 0.694 |
| LDL-C (mg/dL) | 0.05 | 0.245 | 0.04 | 0.388 | 0.32 | 0.111 | 0.08 | 0.066 | 0.06 | 0.172 | 0.11 | **0.014** |
|  |  | |  | |  | |  | |  | |  | |
| ***CETP* rs3764261** | log additive | | dominant | | recessive | | log additive | | dominant | | recessive | |
| Cholesterol (mg/dL) | 0.09 | **0.040** | 0.09 | 0.051 | 0.06 | 0.177 | 0.03 | **0.018** | 0.04 | **0.025** | 0.04 | 0.109 |
| TG (mg/dL) | 0.00 | 0.934 | 0.03 | 0.544 | -0.04 | 0.312 | -0.05 | 0.297 | -0.02 | 0.701 | -0.07 | 0.126 |
| HDL-C (mg/dL) | 0.11 | **0.006** | 0.09 | **0.023** | 0.09 | **0.024** | 0.10 | **1.72x10^-9^** | 0.11 | **1.96x10^-6^** | 0.14 | **2.60x10^-6^** |
| LDL-C (mg/dL) | -0.01 | 0.754 | 0.01 | 0.816 | -0.03 | 0.345 | -0.02 | 0.720 | 0.00 | 0.934 | -0.04 | 0.410 |
|  |  | |  | |  | |  | |  | |  | |
| ***APOE-C1-C4-C2* rs4420638** | log additive | | dominant | | recessive | | log additive | | dominant | | recessive | |
| Cholesterol (mg/dL) | 0.01 | 0.848 | 0.01 | 0.847 | 0.00 | 0.978 | 0.02 | 0.304 | 0.02 | 0.352 | 0.12 | 0.386 |
| TG (mg/dL) | 0.01 | 0.792 | 0.01 | 0.756 | -0.01 | 0.792 | 0.00 | 0.980 | 0.00 | 0.991 | 0.01 | 0.902 |
| HDL-C (mg/dL) | -0.08 | 0.061 | -0.07 | 0.103 | -0.08 | **0.042** | -0.08 | 0.045 | -0.09 | **0.032** | 0.03 | 0.400 |
| LDL-C (mg/dL) | 0.03 | 0.339 | 0.03 | 0.315 | -0.03 | 0.894 | 0.02 | 0.738 | 0.01 | 0.883 | 0.07 | 0.097 |
